# Longitudinal serological measures of common infection in the Avon Longitudinal Study of Parents and Children cohort

**DOI:** 10.12688/wellcomeopenres.14565.2

**Published:** 2018-07-23

**Authors:** Ruth E. Mitchell, Hannah J. Jones, Robert H. Yolken, Glen Ford, Lorraine Jones-Brando, Susan M. Ring, Alix Groom, Sophie FitzGibbon, George Davey Smith, Nicholas J. Timpson

**Affiliations:** 1 Medical Research Council Integrative Epidemiology Unit, Population Health Sciences, Bristol Medical School, University of Bristol, Bristol, UK; 2 Centre for Academic Mental Health, Population Health Sciences, Bristol Medical School, University of Bristol, Bristol, UK; 3 National Institute for Health Research Bristol Biomedical Research Centre, University Hospitals Bristol NHS Foundation Trust and University of Bristol, Bristol, UK; 4 Stanley Division of Developmental Neurovirology, Johns Hopkins University School of Medicine, Baltimore, MD, USA; 5 Population Health Sciences, Bristol Medical School, University of Bristol, Bristol, UK

**Keywords:** Infection, antibody, ALSPAC

## Abstract

Antibodies against pathogens provide information on exposure to infectious agents and are meaningful measures of past and present infection. Antibodies were measured in the plasma of children that are the offspring in a population-based birth cohort, the Avon Longitudinal Study of Parents and Children (ALSPAC). Plasma was collected during clinics at age 5, 7, 11 and 15 years. The antigens examined include: fungal ( *Saccharomyces cerevisiae)*; protozoan ( *Toxoplasma gondii *and surface antigen 1 of *T. gondii)*; herpes viruses (cytomegalovirus, Epstein-Barr virus, herpes simplex virus type 1); common colds (influenza virus subtypes H1N1 and H3N2); other antigens (measles); animal (feline herpes virus, Theiler’s virus); bacteria ( *Helicobacter pylori*); dietary antigens (bovine casein alpha protein, bovine casein beta protein). Alongside the depth of data available within the ALSPAC cohort, this longitudinal resource will enable the investigation of the association between infections and a wide variety of outcomes.

## Introduction

Exposure to pathogens can have a profound impact on the health of an individual both directly and through connections with other diseases ^1^. Studies have linked infectious agents to autoimmune diseases (such as with multiple sclerosis ^2^ and type 1 diabetes in children ^3^), cancers ^4^, drug hypersensitivity ^5^ and psychiatric disorders ^6^. Investigating early life exposure to specific infectious agents is critical to our understanding of the infectious aetiologies of diseases of interest and the impact that infection may have on development.

The Avon Longitudinal Study of Parents and Children (ALSPAC) is a longitudinal birth cohort that recruited pregnant women living near Bristol, UK with an estimated delivery date between 1991 and 1992 ^7, 8^. The study includes extensive phenotypic, genetic, epigenetic and metabolomic data on the mothers, fathers and children, and follow up is ongoing ^9^.

Antibody levels against a wide variety of infections (some not previously measured in humans) have been measured in the plasma of ALSPAC children using ELISA, giving an indication of whether an individual has been exposed to a specific infection or bacteria. The infectious agents studied were chosen based on previous evidence of association with psychiatric outcomes, however they are also of interest in other research domains.

## Methods

A 10% subsample of the ALSPAC cohort (known as “Children in Focus”) were invited to attend research clinics in the early years of the study. From the age of 7 the whole cohort were invited to take part in research clinic assessments.

Blood samples used were taken during the following ALSPAC clinics: 5 years (“Children in Focus” clinic) collected between July 1997 and March 1998; 7 years (“Focus @ 7” clinic) collected between September 1998 and October 2000; 11 years (“Focus 11 +” clinic) collected between January 2003 and January 2005; 15 years (“TeenFocus 3” clinic) collected between October 2006 and November 2008.

Whole blood from each clinic was processed by centrifuging at 3500rpm, 4–5°C for 10 minutes. Plasma was subsequently aliquoted and stored temporarily at -20°C before long term storage at -70/80°C. For samples collected at 15 years plasma was immediately stored at -70/80°C. Samples remained frozen until plated out into 96 well plates for analysis, apart from a subset of samples within the 7 years and 15 years clinics which had undergone 1 previous freeze/thaw cycle before plating.

All available EDTA plasma samples from the 5 years, 7 years and 15 years clinics were plated out. For the 15 years clinic if an EDTA plasma sample was not available from an individual a heparin plasma sample was used if one was available. Only heparin plasma samples were collected at the 11 years clinic and therefore plated out for this time point. If an individual had plasma available from multiple clinics these were aliquoted into the same 96 well plate. Following plating out samples were refrozen before analysis. IgG antibody titers (IgA for *Saccharomyces cerevisiae*) specific for the antigen of the infection of interest in the plasma of ALSPAC children were measured by ELISA using methods derived from those which have been previously described ^10^. Briefly microtiter plates coated with antigens were reacted sequentially with a dilution of human plasma, enzyme labelled anti-human IgG and enzyme substrate with each step separated by plate washing. The amount of colour generated by the ensuing enzyme-substrate reaction was measured by a microplate colourimeter. For some analyses the measured signal was converted to a standardized score with a mean of 2 and a standard deviation of 1 for each plate. The antigens used for the assays are displayed in Supplementary Table 1. 

These results were amalgamated into a dataset in RStudio (R-3.4.1) and saved to Stata format.

## Dataset

The antigens examined are the following: fungal ( *S. cerevisiae)*; protozoan ( *Toxoplasma gondii* and surface antigen 1 (SAG1) of *T. gondii)*; herpes viruses (cytomegalovirus (CMV), Epstein-Barr virus (EBV), herpes simplex virus type 1 (HSV-1)); common colds (influenza virus subtypes H1N1 and H3N2); other antigens (measles); animal (feline herpes virus, Theiler’s virus); bacteria ( *Helicobacter pylori*); dietary antigens (bovine casein alpha protein (α-casein), bovine casein beta protein (β-casein)).

**Figure 1.**
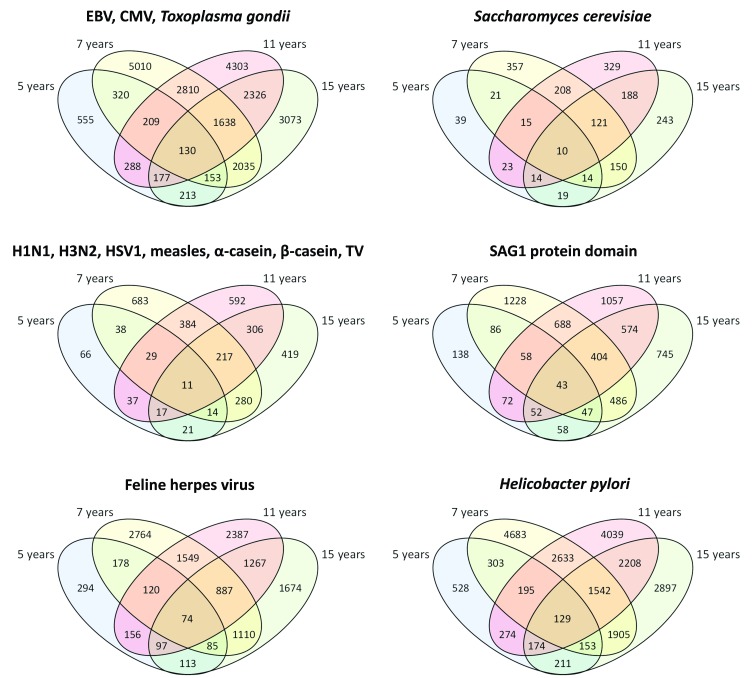
Venn diagram of number of individuals with measures of a specific infection at each combination of ALSPAC clinic ages. Blood samples were taken from ALSPAC participants during clinics at ages: 5 years (“Children in Focus” clinic); 7 years (“Focus @ 7” clinic); 11 years (“Focus 11 +” clinic); 15 years (“TeenFocus 3” clinic). Antibody levels against a wide variety of infections have been measured in the plasma of ALSPAC children using ELISA. Venn diagrams display the combination of the number of individuals with measures across the four time points. **Note:** EBV, Epstein-Barr virus; CMV, cytomegalovirus; Toxo, *Toxoplasma gondii*; H1N1, influenza virus subtype H1N1; H3N2, influenza virus subtype H3N2; HSV1, herpes simplex virus 1; α-casein, alpha-casein protein; β-casein, beta-casein protein; TV, Theiler’s virus.

The ALSPAC plasma samples measured were taken at four ages: 5, 7, 11 and 15 years. The numbers of measures available at each time point for a specific antigen are shown in Table 1. Differences in antibody titer reflect infection history and providing information on past or present exposure to infectious agents. Across the time points, 13909 samples were analysed from 7509 individuals in total (of note: not everyone has been measured at all time points). For each infection three types of measure are provided: the optical density measure read directly from the ELISA plate; the ratio to standards derived from the standards measured on each plate; a standardised z-score for each ratio to standard measure (ratio to standard minus the mean ratio to standard then divided by the standard deviation per plate, plus 2). Across the time points, samples were randomly assigned for the antibodies measured. The combination of the number of individuals with antibody titer measures across the four time points are shown in the Venn diagrams in Figure 1 and Supplementary Table 2. This helps to demonstrate the breadth and depth of longitudinal research that is possible with the dataset in terms of power.

**Table 1.** Number of measures of a specific infection (with mean age in months and percentage female) available from each ALSPAC clinic. Blood samples were taken from ALSPAC participants during clinics at ages: 5 years (“Children in Focus” clinic); 7 years (“Focus @ 7” clinic); 11 years (“Focus 11 +” clinic); 15 years (“TeenFocus 3” clinic). Antibody levels against a wide variety of infections have been measured in the plasma of ALSPAC children using ELISA. This table details the numbers of individuals measured at each time point for a specific antigen.

	ALSPAC clinic N (mean age in months [sd]; % female)				
Antigen	5 year clinic	7 year clinic	11 year clinic	15 year clinic	Total N
Total samples sent over	594 (61.87 [0.77]; 43.1%)	5367 (90.49 [3.91]; 48.46%)	4632 (140.92 [2.88]; 51.19%)	3316 (185.04, [3.1]; 50.87%)	13,909
*Saccharomyces* *cerevisiae*	39 (61.79 [0.7]; 43.59%)	357 (90.4 [3.74]; 51.26%)	329 (140.62 [2.73]; 55.02%)	243 (185.11, [3.03]; 53.91%)	968
*Toxoplasma gondii*	555 (61.88 [0.78]; 43.06%)	5010 (90.49 [3.92]; 48.26%)	4303 (140.94 [2.89]; 50.89%)	3073 (185.04, [3.11]; 50.63%)	12,941
SAG1 protein domain	138 (61.8 [0.67]; 39.86%)	1228 (90.38 [3.87]; 46.91%)	1057 (141 [2.92]; 48.82%)	745 (185.16, [3.27]; 46.71%)	3168
Cytomegalovirus	555 (61.88 [0.78]; 43.06%)	5010 (90.49 [3.92]; 48.26%)	4303 (140.94 [2.89]; 50.89%)	3073 (185.04, [3.11]; 50.63%)	12,941
Epstein-Barr virus	555 (61.88 [0.78]; 43.06%)	5010 (90.49 [3.92]; 48.26%)	4303 (140.94 [2.89]; 50.89%)	3073 (185.04, [3.11]; 50.63%)	12,941
Herpes simplex virus 1	66 (61.82 [0.84]; 50%)	683 (90.63 [3.97]; 49.63%)	592 (140.83 [2.91]; 52.2%)	419 (184.91, [3.09]; 53.46%)	1760
Influenza virus subtype H1N1	66 (61.82 [0.84]; 50%)	683 (90.63 [3.97]; 49.63%)	592 (140.83 [2.91]; 52.2%)	419 (184.91, [3.09]; 53.46%)	1760
Influenza virus subtype H3N2	66 (61.82 [0.84]; 50%)	683 (90.63 [3.97]; 49.63%)	592 (140.83 [2.91]; 52.2%)	419 (184.91, [3.09]; 53.46%)	1760
Measles virus	66 (61.82 [0.84]; 50%)	683 (90.63 [3.97]; 49.63%)	592 (140.83 [2.91]; 52.2%)	419 (184.91, [3.09]; 53.46%)	1760
Feline herpes virus	294 (61.84 [0.8]; 43.2%)	2764 (90.48 [3.91]; 47.5%)	2387 (140.94 [2.86]; 49.77%)	1674 (185.1, [3.16]; 49.04%)	7119
Theiler's virus	66 (61.82 [0.84]; 50%)	683 (90.63 [3.97]; 49.63%)	592 (140.83 [2.91]; 52.2%)	419 (184.91, [3.09]; 53.46%)	1760
*Helicobacter pylori*	528 (61.88 [0.77]; 42.23%)	4683 (90.46 [3.9]; 48.3%)	4039 (140.93 [2.87]; 51.05%)	2897 (185.06, [3.11]; 50.5%)	12,147
Alpha-casein protein	66 (61.82 [0.84]; 50%)	683 (90.63 [3.97]; 49.63%)	592 (140.83 [2.91]; 52.2%)	419 (184.91, [3.09]; 53.46%)	1760
Beta-casein protein	66 (61.82 [0.84]; 50%)	683 (90.63 [3.97]; 49.63%)	592 (140.83 [2.91]; 52.2%)	419 (184.91, [3.09]; 53.46%)	1760

As an initial pilot study, levels of antibodies to CMV, *T. gondii*, HSV-1, measles and whole casein antigen (a mixture of α-casein and β-casein) were measured in a random subset of samples (968 samples from 513 individuals). The sources of antigen used in this subset are distinct from the main sample and therefore it is recommended to analyse the results of this subset separately from the main sample as the antigens used are not directly comparable. The numbers of measures available at each time point for a specific antigen in this subset are shown in Supplementary Table 3 and the combinations of number of individuals with measures are shown in the Venn diagram in Supplementary Figure 1.

**Figure 2.**
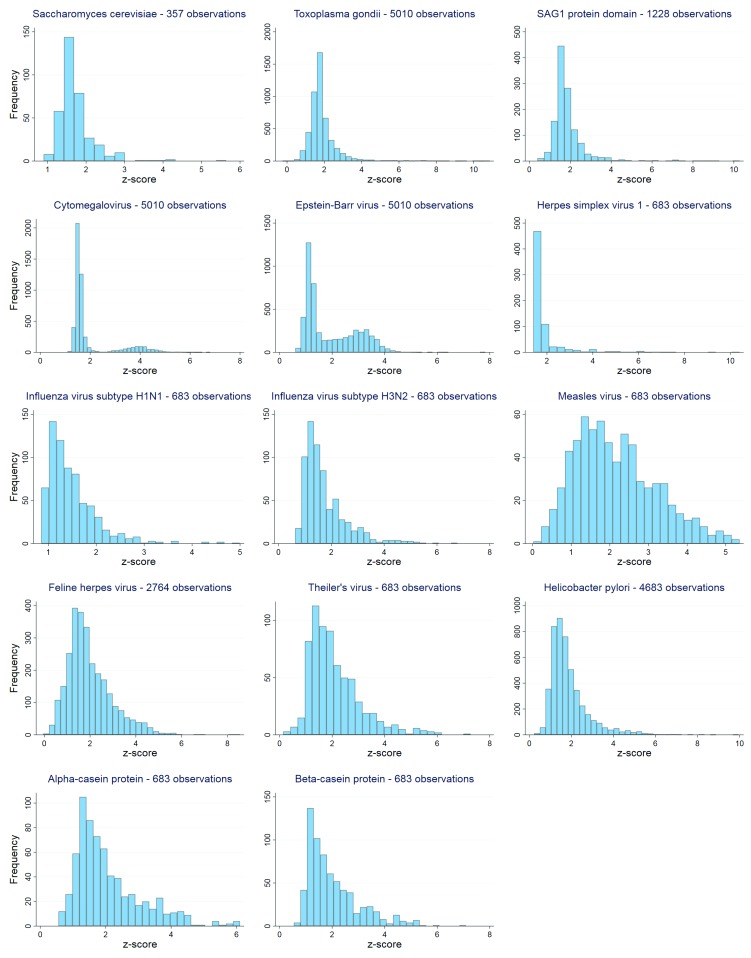
Z-score distributions of the different infections at 7 year ALSPAC clinic. Blood samples were taken from ALSPAC participants during the clinic at age 7 years (“Focus @ 7” clinic). Antibody levels against a wide variety of infections have been measured in the plasma of ALSPAC children using ELISA. Graphs show the distribution of the standardised z-score for the ratio to standard measure (ratio to standard minus the mean ratio to standard then divided by the standard deviation per plate, plus 2) for each antigen measured in the plasma taken at the 7 year clinic.

The distributions of the standardised z-scores of the infections at the ALSPAC 7 year clinic are displayed in Figure 2. The distributions of infections at other clinic ages are shown in Supplementary Figure 2– Supplementary Figure 4. CMV and EBV show a bimodal distribution at all ages. To aid in interpretation, cut-offs may be taken for these infections dividing them into two categories: ‘positive’ and ‘negative’, or three categories: ‘positive’, ‘in between’ and ‘negative’. Measles and *T. gondii* and its surface protein SAG1 have normal distributions across the samples. In the case of measles this may be due to viral exposure through vaccine.

Several animal (zoonotic) viruses have been measured. The measure of feline herpes virus and the murine Theiler’s virus is novel in humans and both show a distribution with a positive skew. IgG antibodies to casein proteins, the family of proteins in cow’s milk, can mirror an autoimmune response and has been associated with gastrointestinal inflammation.

Antibodies against influenza (H1N1 and H3N2) and the bacteria *H. pylori* display a distribution with a large positive skew. The yeast *S. cerevisiae* displays different distribution at different ages.

The dataset has excellent longitudinal data over four time points in this birth cohort. Due to the deep phenotype and genetic data in ALSPAC, this dataset provides the opportunity for a wide range of epidemiological and genetic analysis.

## Ethical approval and consent

Ethical approval for the study was obtained from the ALSPAC Ethics and Law Committee and the Local Research Ethics Committees, full details of the approvals obtained are available from the study website ( http://www.bristol.ac.uk/alspac/researchers/research-ethics/).

Written informed consent to take and analyse blood samples was obtained from the parents of participating children at each clinic visit. Children were invited to give assent where appropriate. Study members have the right to withdraw their consent for elements of the study or from the study entirely at any time.

## Data availability

ALSPAC data access is through a system of managed open access. The steps below highlight how to apply for access to the data included in this data note and all other ALSPAC data. The ALSPAC variable codes highlighted in the dataset descriptions can be used to specify required variables.

1. Please read the ALSPAC access policy (PDF, 627kB) which describes the process of accessing the data and samples in detail, and outlines the costs associated with doing so.

2. You may also find it useful to browse our fully searchable research proposals database, which lists all research projects that have been approved since April 2011.

3. Please submit your research proposal for consideration by the ALSPAC Executive Committee using the online process. You will receive a response within 10 working days to advise you whether your proposal has been approved.

If you have any questions about accessing data, please email alspac-data@bristol.ac.uk.

The ALSPAC data management plan describes in detail the policy regarding data sharing, which is through a system of managed open access.

## Supplementary material

**Supplementary File 1. Supplementary Tables (1–3).**

**Supplementary Figure 1. Venn diagram of number of individuals with measures at each combination of ALSPAC clinic ages for the subset of ALSPAC individuals measured using different sources of antigens as compared to main dataset.** Blood samples were taken from ALSPAC participants during clinics at ages: 5 years (“Children in Focus” clinic); 7 years (“Focus @ 7” clinic); 11 years (“Focus 11 +” clinic); 15 years (“TeenFocus 3” clinic). This ALSPAC infection subset contains antibody levels against *Toxoplasma gondii*, cytomegalovirus, herpes simplex virus 1, measles virus and casein protein (a mixture of casein alpha and casein beta protein) only.

**Supplementary Figure 2. Z-score distributions of the different infections at age 5 years.** Blood samples were taken from ALSPAC participants during the clinic at age 5 years (“Children in Focus” clinic). Antibody levels against a wide variety of infections have been measured in the plasma of ALSPAC children using ELISA. Graphs show the distribution of the standardised z-score for the ratio to standard measure (ratio to standard minus the mean ratio to standard then divided by the standard deviation per plate, plus 2) for each antigen measured in the plasma taken at the 5 year clinic.

**Supplementary Figure 3. Z-score distributions of the different infections at age 11 years.** Blood samples were taken from ALSPAC participants during the clinic at age 11 years (“Focus 11 +” clinic). Antibody levels against a wide variety of infections have been measured in the plasma of ALSPAC children using ELISA. Graphs show the distribution of the standardised z-score for the ratio to standard measure (ratio to standard minus the mean ratio to standard then divided by the standard deviation per plate, plus 2) for each antigen measured in the plasma taken at the 11 year clinic.

**Supplementary Figure 4. Z-score distributions of the different infections at age 15 years.** Blood samples were taken from ALSPAC participants during the clinic at 15 years (“TeenFocus 3” clinic). Antibody levels against a wide variety of infections have been measured in the plasma of ALSPAC children using ELISA. Graphs show the distribution of the standardised z-score for the ratio to standard measure (ratio to standard minus the mean ratio to standard then divided by the standard deviation per plate, plus 2) for each antigen measured in the plasma taken at the 15 year clinic.
