# Supplementary material for: Longitudinal serological measures of common infection in the Avon Longitudinal Study of Parents and Children cohort
Source: Wellcome Open Res. 2018 Jul 23;3:49. Originally published 2018 Apr 27. [Version 2] doi: 10.12688/wellcomeopenres.14565.2 (PMC6124408; doi:10.12688/wellcomeopenres.14565.2)
Supplement: Supplementary file 1 [file wellcomeopenres-3-16026-s0000.tgz › 1d438e18-7869-4c0a-9724-48c09c0a4094.docx]

**Supplementary File 1: Supplementary tables (1-3).**

**Supplementary Table 1. Source of antigen used to measure each infection. Note:** H1N1, influenza virus subtype H1N1; H3N2, influenza virus subtype H3N2; EBV, Epstein-Barr virus; HSV1, herpes simplex virus 1; CMV, cytomegalovirus.

| **Target antigen** | **Source of antigen** |
| --- | --- |
| H1N1 recombinant protein | eENZYME (catalogue number: IA 0012W) |
| H3N2 recombinant protein | eENZYME (catalogue number: 1AH3-050) |
| EBV whole virus lysate | Meridian Life Sciences (catalogue number: 7420) |
| HSV1 gg1 recombinant protein | Meridian Life Sciences (catalogue number: VTI-520) |
| *Toxoplasma gondii* whole tachyzoites | Ross Southern Lab (catalogue number: NF1011) |
| SAG-1 recombinant protein | Devetal Labs (catalogue number: 2604) |
| CMV whole virus lysate | Virusys (catalogue number: CV08Y) |
| Measles whole virus lysate | Fitzgerald Industries (catalogue number: 30-1308) |
| Feline Herpes Virus lysate | Ross Southern Lab (catalogue number: NF5808) |
| Theiler’s Murine Virus recombinant | Fitzgerald Industries (catalogue number: 30-1269) |
| Alpha Casein purified protein | Sigma (catalogue number: C6780) |
| Beta casein purified protein | Sigma (catalogue number: 6905) |
| *Saccharomyces cerevisiae* type 1 and 2 | Sigma (catalogue number: YSC1; YSC2) |
| *Helicobacter pylori* whole lysate | Meridian Life Sciences (catalogue number: RS2101) |

**Supplementary Table 2. The combination of the number of individuals with antibody titer measures across the four time points.** Blood samples were taken from ALSPAC participants during clinics at ages: 5 years (“Children in Focus” clinic); 7 years (“Focus @ 7” clinic); 11 years (“Focus 11 +” clinic); 15 years (“TeenFocus 3” clinic). Antibody levels against a wide variety of infections have been measured in the plasma of ALSPAC children using ELISA. Table display the combination of the number of individuals with measures across the four time points.

|  | **Epstein-Barr virus, Cytomegalovirus, *Toxoplasma gondii*** | | | | ***Saccharomyces cerevisiae*** | | | |
| --- | --- | --- | --- | --- | --- | --- | --- | --- |
| **Clinic ages (years)** | **5** | **7** | **11** | **15** | **5** | **7** | **11** | **15** |
| **5** | 555 | - | - | - | 39 | - | - | - |
| **7** | 320 | 5010 | - | - | 21 | 357 | - | - |
| **11** | 288 | 2810 | 4303 | - | 23 | 208 | 329 | - |
| **15** | 213 | 2035 | 2326 | 3073 | 19 | 150 | 188 | 243 |
| **7 and 11** | 209 | 2810 | 2810 | 1638 | 15 | 208 | 208 | 121 |
| **7 and 15** | 153 | 2035 | 1638 | 2035 | 14 | 150 | 121 | 150 |
| **11 and 15** | 177 | 1638 | 2326 | 2326 | 14 | 121 | 188 | 188 |
| **5, 7, 11 and 15** | 130 | | | | 10 | | | |
|  | **Influenza virus subtypes H1N1 and H3N2, Herpes simplex virus 1, Measles virus,**  **Alpha-casein protein, Beta-casein protein, Theiler's virus** | | | | **SAG1 protein domain** | | | |
| **Clinic ages (years)** | **5** | **7** | **11** | **15** | **5** | **7** | **11** | **15** |
| **5** | 66 | - | - | - | 138 | - | - | - |
| **7** | 38 | 683 | - | - | 86 | 1228 | - | - |
| **11** | 37 | 384 | 592 | - | 72 | 688 | 1057 | - |
| **15** | 21 | 280 | 306 | 419 | 58 | 486 | 574 | 745 |
| **7 and 11** | 29 | 384 | 384 | 217 | 58 | 688 | 688 | 404 |
| **7 and 15** | 14 | 280 | 217 | 280 | 47 | 486 | 404 | 486 |
| **11 and 15** | 17 | 217 | 306 | 306 | 52 | 404 | 574 | 574 |
| **5, 7, 11 and 15** | 11 | | | | 43 | | | |
|  | **Feline herpes virus** | | | | ***Helicobacter pylori*** | | | |
| **Clinic ages (years)** | **5** | **7** | **11** | **15** | **5** | **7** | **11** | **15** |
| **5** | 294 | - | - | - | 528 | - | - | - |
| **7** | 178 | 2764 | - | - | 303 | 4683 | - | - |
| **11** | 156 | 1549 | 2387 | - | 274 | 2633 | 4039 | - |
| **15** | 113 | 1110 | 1267 | 1674 | 211 | 1905 | 2208 | 2897 |
| **7 and 11** | 120 | 1549 | 1549 | 887 | 195 | 2633 | 2633 | 1542 |
| **7 and 15** | 85 | 1110 | 887 | 1110 | 153 | 1905 | 1542 | 1905 |
| **11 and 15** | 97 | 887 | 1267 | 1267 | 174 | 1542 | 2208 | 2208 |
| **5, 7, 11 and 15** | 74 | | | | 129 | | | |

**Supplementary Table 3. Number of measures (with mean age in months and percentage female) available in the subset of ALSPAC individuals measured using different sources of antigens as compared to main dataset.** Blood samples were taken from ALSPAC participants during clinics at ages: 5 years (“Children in Focus” clinic); 7 years (“Focus @ 7” clinic); 11 years (“Focus 11 +” clinic); 15 years (“TeenFocus 3” clinic). This ALSPAC infection subset contains antibody levels against *Toxoplasma gondii*, cytomegalovirus, herpes simplex virus 1, measles virus and casein protein (a mixture of casein alpha and casein beta protein) only.

| **ALSPAC clinic subset N**  **(mean age in months [sd]; % female)** | | | |  |
| --- | --- | --- | --- | --- |
| **5 year clinic** | **7 year clinic** | **11 year clinic** | **15 year clinic** | **Total N** |
| 39  (61.79 [0.70];  43.59%) | 357  (90.40 [3.74];  51.26%) | 329  (140.62 [2.73]; 55.02%) | 243  (185.11, [3.03];  53.91%) | 968 |
