# Supplementary figures and images for: Longitudinal serological measures of common infection in the Avon Longitudinal Study of Parents and Children cohort
Source: Wellcome Open Res. 2018 Jul 23;3:49. Originally published 2018 Apr 27. [Version 2] doi: 10.12688/wellcomeopenres.14565.2 (PMC6124408; doi:10.12688/wellcomeopenres.14565.2)

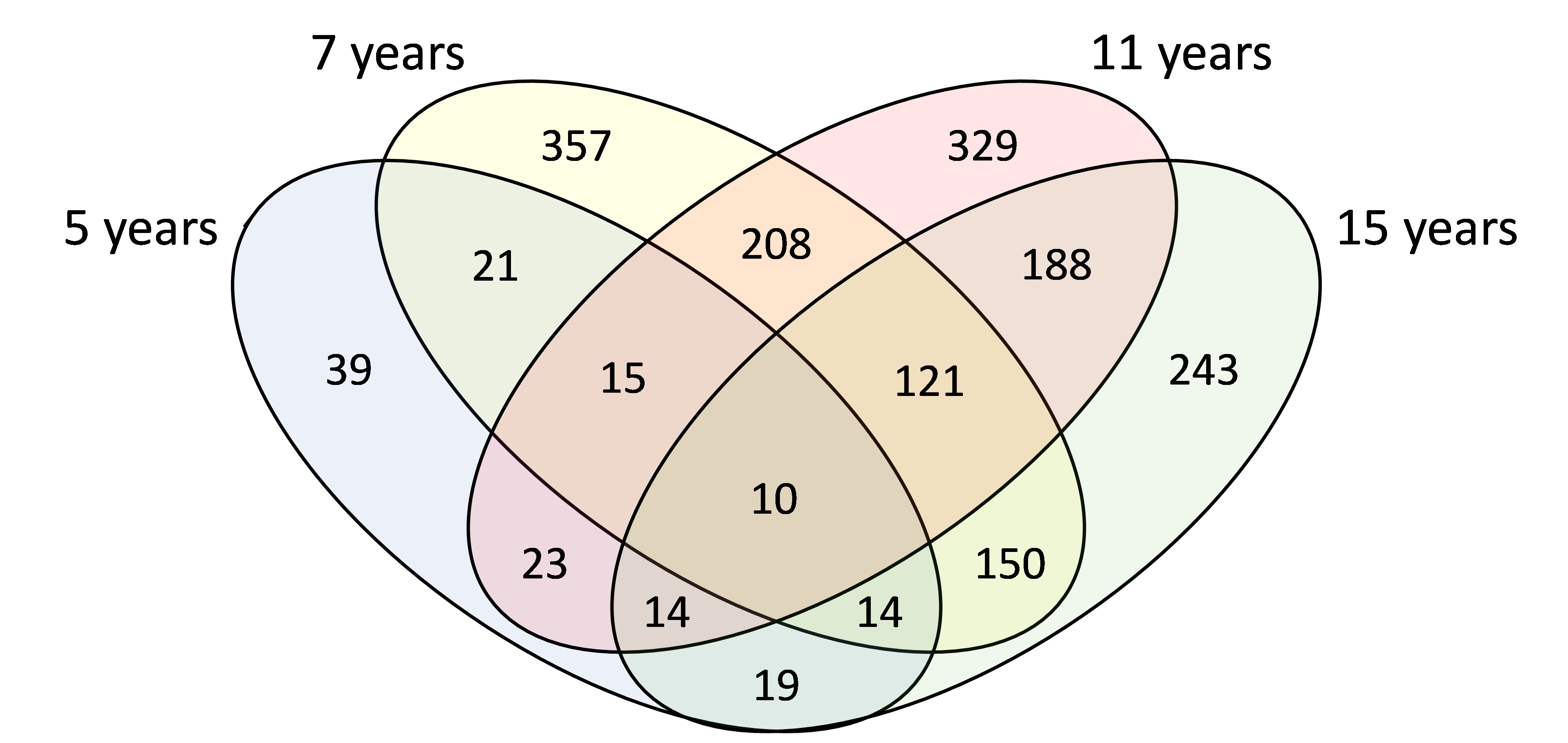

Supplement: Supplementary file 2 [file wellcomeopenres-3-16026-s0001.tgz › 7ff48936-06df-48bb-bd5d-4c44a3b7b341.jpg]

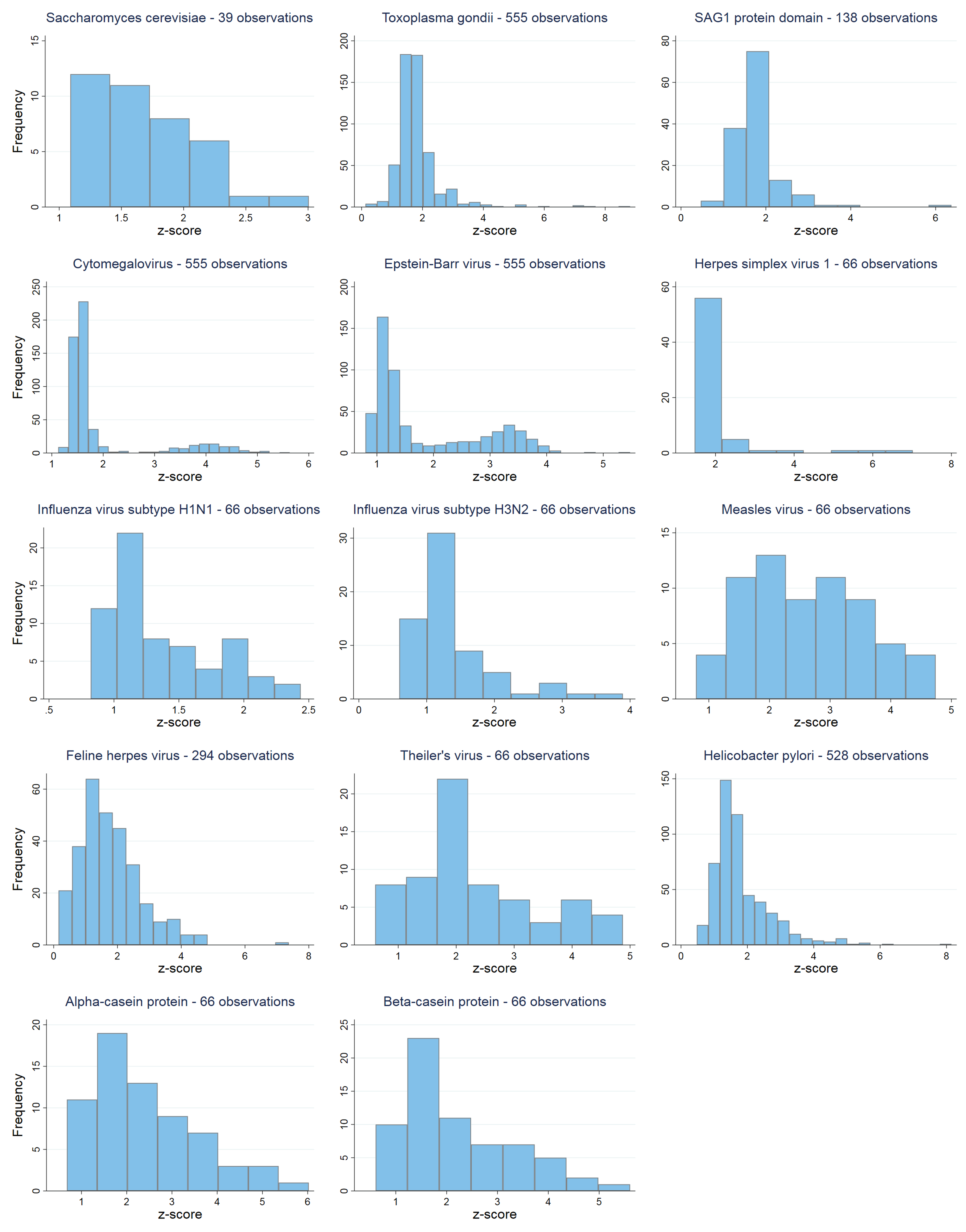

Supplement: Supplementary file 3 [file wellcomeopenres-3-16026-s0002.tgz › 1a53eefd-a902-49f5-90b0-ced3a3638b6b.png]

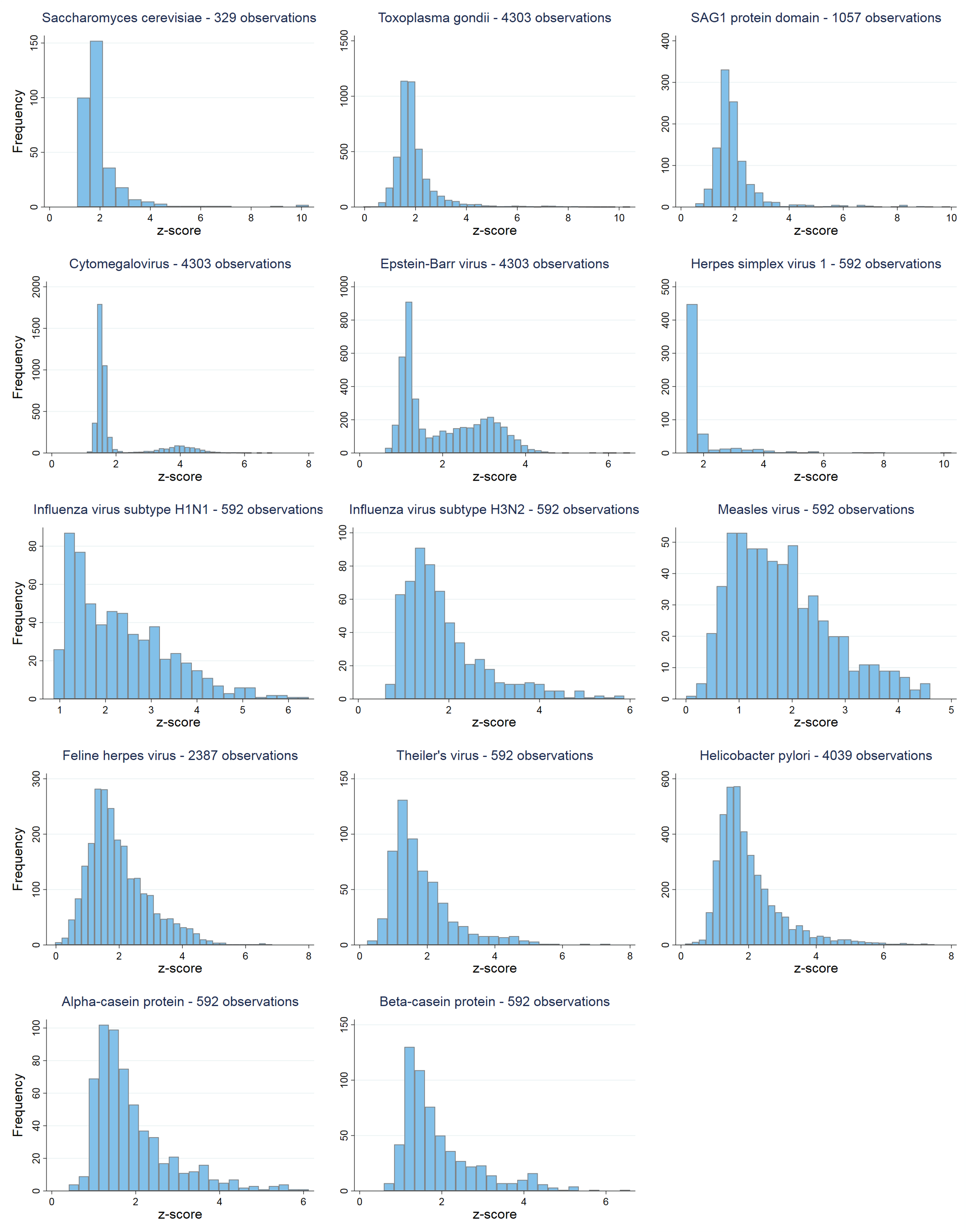

Supplement: Supplementary file 4 [file wellcomeopenres-3-16026-s0003.tgz › 86a16d21-15a9-4517-8c52-6e5f1d23bd6d.png]

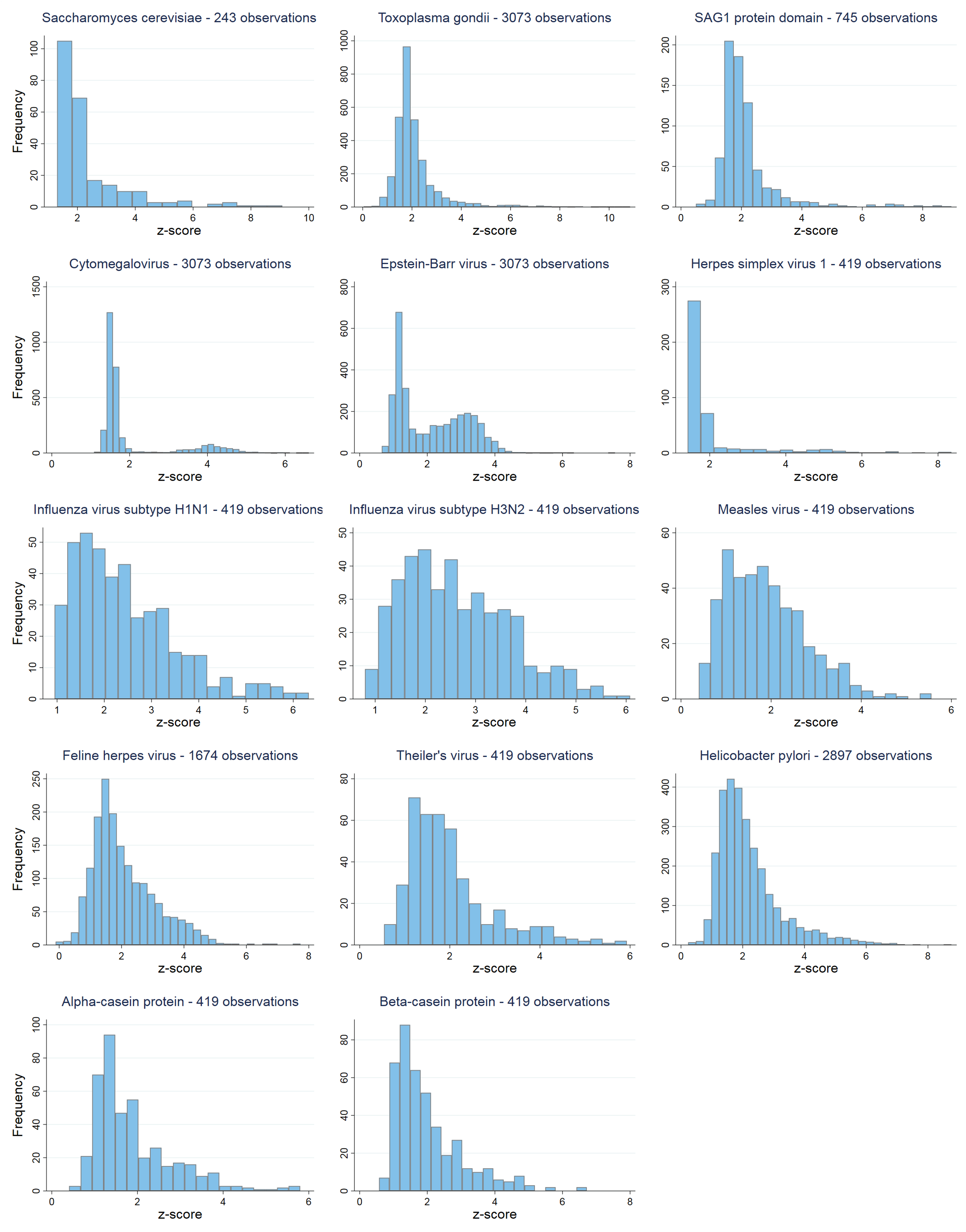

Supplement: Supplementary file 5 [file wellcomeopenres-3-16026-s0004.tgz › a4d3a722-a41c-4747-91d1-05683c554d0e.png]
