# Supplementary material for: Longitudinal serological measures of common infection in the Avon Longitudinal Study of Parents and Children cohort
Source: Wellcome Open Res. 2018 Jul 23;3:49. Originally published 2018 Apr 27. [Version 2] doi: 10.12688/wellcomeopenres.14565.2 (PMC6124408; doi:10.12688/wellcomeopenres.14565.2)
Supplement: Peer review history

# Peer review: Referee response for version 2

**DOI:** 10.21956/wellcomeopenres.16026.r33735

**Published:** 2018-09-04

**Authors:** David Burgner

**Affiliations:** 1 Royal Children's Hospital, Murdoch Children's Research Institute, Parkville, Vic, Australia; 2 University of Melbourne, Parkville, Vic, Australia

This is an important and interesting data note of a very well characterised cohort. 

 Even though this is a data note, it would be helpful to have a text box or table briefly outlining the rationale for the choice of each of the antigens to provide some scientific and clinical context. I am not sure 'common colds' is the best descriptor for specific influenza A infections.

 In addition to the Venn diagrams, it would be interesting to present data on the change in intra-individual titres at successive time points.

 In Table 1, it is unclear what the row heading 'total samples sent over' refers to. Is this the samples sent to the lab for analyses?

I have read this submission. I believe that I have an appropriate level of expertise to confirm that it is of an acceptable scientific standard.

# Peer review: Referee response for version 1

**DOI:** 10.21956/wellcomeopenres.15853.r33187

**Published:** 2018-06-27

**Authors:** Allison E. Aiello, Rebecca Stebbins

**Affiliations:** 1 Department of Epidemiology, Gillings School of Global Public Health, Carolina Population Center, University of North Carolina at Chapel Hill, Chapel Hill, NC, USA; 2 University of North Carolina, Chapel Hill, NC, USA

This is a well-described description of the laboratory testing used to generate the results for several pathogens in the ALSPAC study. The existing ALSPAC data is described and a clear rationale for adding the novel pathogen data, has been provided. Specifically, the authors provide a sufficient level of detail on the methods for collecting the samples, storage, and laboratory testing of pathogens. We identified one paragraph that requires some clarification: 

 Referring to the last paragraph on page 3 - The authors discuss a “subset of samples (968 samples from 513 individuals)” for which the authors recommend conducting a separate analysis. It would be helpful to have further information on how this subset was selected and why it should be analyzed separately from the overall cohort.

We have read this submission. We believe that we have an appropriate level of expertise to confirm that it is of an acceptable scientific standard.

# Response

**Published:** 2018-07-18

**Authors:** Ruth Mitchell

**Affiliations:** University of Bristol, UK

We thank the referee for their helpful comments.

 We have edited the manuscript to provide clarity about why we recommend a subset of samples to be analysed separately. “As an initial pilot study, levels of antibodies to CMV, *T. gondii*, HSV-1, measles and whole casein antigen (a mixture of α-casein and β-casein) were measured in a random subset of samples (968 samples from 513 individuals). The sources of antigen used in this subset are distinct from the main sample and therefore it is recommended to analyse the results of this subset separately from the main sample as the antigens used are not directly comparable.”
